# Deep Learning-Based Dynamic Region of Interest Autofocus Method for Grayscale Image

**DOI:** 10.3390/s24134336

**Published:** 2024-07-04

**Authors:** Yao Wang, Chuan Wu, Yunlong Gao, Huiying Liu

**Affiliations:** 1 Changchun Institute of Optics, Fine Mechanics and Physics, Chinese Academy of Sciences, Changchun 130033, China; wangyao222@mails.ucas.ac.cn (Y.W.); gaoyl15@mails.jlu.edu.cn (Y.G.); liuhuiying21@mails.ucas.ac.cn (H.L.); 2 University of Chinese Academy of Sciences, Beijing 100049, China

**Keywords:** autofocus, dataset, deep learning, lightweight network, ordinal regression

## Abstract

In the field of autofocus for optical systems, although passive focusing methods are widely used due to their cost-effectiveness, fixed focusing windows and evaluation functions in certain scenarios can still lead to focusing failures. Additionally, the lack of datasets limits the extensive research of deep learning methods. In this work, we propose a neural network autofocus method with the capability of dynamically selecting the region of interest (ROI). Our main work is as follows: first, we construct a dataset for automatic focusing of grayscale images; second, we transform the autofocus issue into an ordinal regression problem and propose two focusing strategies: full-stack search and single-frame prediction; and third, we construct a MobileViT network with a linear self-attention mechanism to achieve automatic focusing on dynamic regions of interest. The effectiveness of the proposed focusing method is verified through experiments, and the results show that the focusing MAE of the full-stack search can be as low as 0.094, with a focusing time of 27.8 ms, and the focusing MAE of the single-frame prediction can be as low as 0.142, with a focusing time of 27.5 ms.

## 1. Introduction

Autofocus technology holds significant application value in both military and civilian fields, particularly playing a key role in scenarios that require rapid and precise target capture. Autofocus methods for optical systems can generally be divided into two main categories: active focusing and passive focusing. Active focusing methods often rely on external sensors, which undoubtedly increase the manufacturing cost and technical complexity of the optical system. In contrast, passive focusing is based on image quality to provide feedback for focus control.

Real-world scenarios consist of a three-dimensional space containing multiple objects. For a given optical lens, there is a certain depth of field range, which means that within the same scene, the lens can only keep a portion of the targets within a finite depth of field. Regarding the selection of the focusing window, there are currently two main solutions: the first is a user-interactive selection of the focusing window, and the second is a fixed template focusing window, which is determined in advance using prior knowledge [1]. The two methods are not without their individual shortcomings.

**Figure 1:**
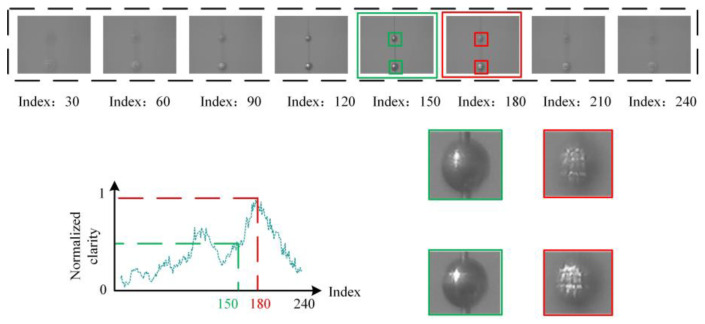
Misjudgment of light spots. Most evaluation functions tend to misjudge in bokeh scenes, where the red box indicates the misjudged images and the green box represents the actual clear images.

After the focusing window is determined, traditional autofocus methods assess image quality through fixed convolutional kernels or methods in the transform domain, ultimately bringing the target within the area into focus. However, there are two insurmountable issues with traditional image-based autofocus solutions. The first issue is the misjudgment of light spots. The reason for this is that the point spread function (PSF) in a focused state is more concentrated with high intensity values, whereas in a defocused state, the PSF is more dispersed with lower intensity values. When taking the gradient of these two states, the latter may have a greater advantage. As shown in Figure 1, the example uses the Laplacian as a sharpness evaluation function, with the vertical axis representing the normalized evaluation value and the horizontal axis representing the image number. It can be observed that the defocused state contains more gradient energy than the focused state, which can lead to a misjudgment of the focus value by the sharpness evaluation function.

**Figure 2:**
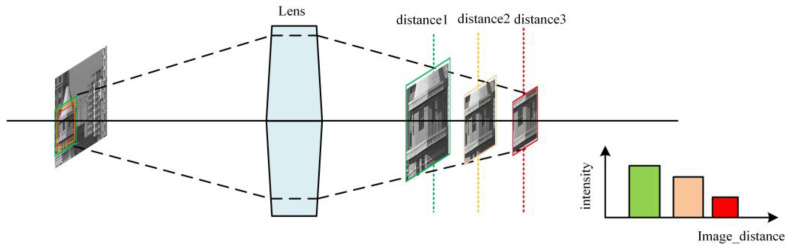
Different colors correspond to images with different border frames in the figure.

The second issue is focal breathing [2]. As shown in Figure 2, since the camera’s focusing process is achieved by changing the distance between the imaging plane and the lens, this also causes certain changes in the camera’s zoom motor. This results in changes to the boundary information entering the focusing window during the focusing process, even in a static image. Additionally, the light intensity at different distances will also undergo slight variations. Both of these reasons can cause fluctuations in the image sharpness evaluation function curve that are not due to changes in image quality.

With the advent of the artificial intelligence era, neural networks have achieved significant results in image classification, semantic segmentation, and object detection, with their ability to extract high-dimensional image features and understand images far surpassing traditional methods [3,4,5]. In terms of image feature extraction, neural networks can effectively extract the main subjects within a scene, and the feature maps are primarily derived from the contribution of the main subjects [6]; for depth estimation, neural networks can estimate the relative distances of various objects in the scene from monocular images, thereby obtaining depth information [7,8].

Inspired by the widespread application of deep learning in computer vision tasks, applying neural networks to the field of autofocus can also yield satisfactory results. In recent years, researchers have applied convolutional networks to the field of autofocus. To expand the dataset and simulate user focus selection, researchers segmented the complete image data into sequences of images of the same size, turning image data into a focus stack [2]. We further optimize the focusing strategy, aiming to construct an end-to-end autofocus network that can better understand the user’s intention in photography.

Our contributions are summarized as follows:

1. We construct an autofocus dataset. We collect multiple sets of image sequences with continuous focusing adjustments in real-world scenarios, which include simple scenes, complex scenes, scenes at long focal lengths, and scenes at short focal lengths. We further expand the dataset using the sliding window method.
2. We convert the autofocus issue into ordinal regression problems, namely full-stack searching and single-frame prediction. Ordinal regression stands as an intermediate challenge between classification and regression. Unlike traditional focusing strategies, our focusing method does not require the determination of the focusing area in advance; the network adaptively focuses on the region of interest in the frame. We investigate the effects of traditional ordinal regression loss functions on the training of neural networks through our experiments.
3. We introduce a linear self-attention module into the MobileViT network, enabling the network to achieve focusing functionality with less computational cost and a more concentrated salient target area extraction capability.

## 2. Related Work

### 2.1. Traditional AF

Most of the research on autofocus is based on traditional methods, which are divided into active focusing and passive focusing [5]. For passive focusing, which is based on images, the research mainly focuses on the following two aspects: (1) the selection of the focusing window and (2) the sharpness evaluation function. A specific sharpness evaluation function is used to assess image quality, and the task of autofocus is to find the point where this indicator is maximized.

The selection of the focusing window was mostly carried out using fixed template windows or through human–computer interaction. Some scholars, from the perspective of photographic art, integrated a prior knowledge into the design of the focusing area, proposing a focusing area selection strategy [1]. They suggested using mid-frequency discrete cosine transform focus measurement to detect objects distributed in the image, and then selecting the target object through fuzzy inference with three fuzzy membership functions. This method is not sensitive to Gaussian noise and impulse noise and can improve image quality by focusing on the appropriate target object [3]. We hope to utilize the powerful feature extraction capabilities of neural networks to integrate the selection of the focusing point into the neural network as well, eliminating the need for a separate step of focus selection.

There has been extensive research on image evaluation functions, mainly based on gradient operators [5,6,7,8], contrast-based [9], entropy-based [10], and transform domain-based methods [11,12,13]. Most of these studies can solve the problem well for small sample data but often lack universality. To address the issue of low contrast, some researchers proposed a new contrast measurement method that avoids local false peaks and ensures the presence of a well-defined focusing peak [14]. In addition to traditional image evaluation functions, there have also been other image evaluation strategies emerging in recent years. For example, some researchers used a combination of threshold gradient and edge point counting techniques with traditional evaluation functions to assess image quality, and finally implemented autofocus using a two-stage hill-climbing search algorithm [15]. There are also researchers who have achieved rough focusing based on the disparity information of two images, bringing the lens to the hill region of the focusing profile in one go [16]. 

The shortcomings of traditional methods are that the focus window cannot be efficiently selected, and the evaluation function cannot adapt to various complex focusing scenarios. Therefore, more and more scholars are trying to use deep learning to solve the problem of autofocus.

### 2.2. ML-Based AF

Initially, researchers compared heuristic methods based on machine learning [17] with previously proposed handcrafted heuristic methods for autofocus and other baseline methods, achieving good results. In recent years, with the rapid development of deep learning in the field of computer vision, the exploration of applying deep learning to autofocus tasks has gradually increased and has been verified in practical applications [18]. Currently, there are mainly two strategies for applying deep learning to the field of autofocus. The first strategy is to optimize a certain part of the traditional method, such as using deep learning to adaptively find the focus point or using deep learning as a method for image quality evaluation. Wang et al. proposed a simple and effective framework using deep convolutional neural networks (DCNN) and transformers for image quality assessment [19]. The second strategy is to adopt an end-to-end focusing approach. There are different views among scholars on the input for end-to-end focusing methods, mainly divided into three types: transform domain input [20], dual-pixel image stack input [2], and arbitrary single blurred image input [21]. For example, Jiang et al. proposed using spatial images, Fourier spectra, and the autocorrelation of spatial images and their combinations as inputs for CNNs, where information from the transform domain can improve the performance and robustness of the autofocus process [22]. Wang et al. for different scenarios, proposed a rule-based agent and a learning agent, achieving autofocus through a closed-loop iterative method of focus selection, image classification, focus value prediction, and image fusion, demonstrating the advantages of this method over other focus stacking methods [23]. Some researchers also proposed a non-iterative unsupervised autofocus scheme based on deep learning and the minimum entropy criterion using entropy as the loss function [24]. In addition, refocusing of a single blurred image can also be achieved through generative networks [25]. 

Using deep learning to address the problem of autofocus is a strategy with many approaches, and the search for the most efficient method is ongoing. In this paper, we aim to adopt an end-to-end strategy to tackle the problem of autofocus. The end-to-end approach is reflected in two main aspects: First, we minimize the processing of the source data as much as possible. In this paper, we use a data stack as the network input without any special preprocessing. The reason for not using a single image in single-frame prediction is that we are concerned the neural network may not be able to determine the directionality of the true focus position from a single image lacking channel information. Second, there is no need to pre-select a focusing window. We hope to leverage the neural network’s feature extraction capabilities to automatically converge attention on salient targets.

### 2.3. Ordinal Regression

Research on ordinal regression problems is relatively scarce, and it is currently mainly focused on applications in age estimation [26]. The main reason for proposing this problem is that for classification networks, the traditional cross-entropy loss uses one-hot encoding as labels. For misclassifications that are at different distances from the truth, the traditional loss cannot provide different levels of punishment related to the order. However, ordinal regression problems have a relative order relationship between labels [27]. For example, in age estimation problems, we hope that our estimation results are a discrete integer value of 25, not 25.5, and when the predicted result is 24 or 26, the loss should be less than when the predicted result is 23 or 27. Ordinal regression is an intermediate problem between classification and regression problems. It allows us to predict the ordered categorical labels of samples based on input features and provides more fine-grained information for decision-making. Ordinal regression, by introducing appropriate models and strategies, allows for more flexible category distribution and different intervals between different categories. For example, Niu et al. transformed ordinal classification tasks into binary classification subtasks [28], and the independent binary classification tasks have the capability of ordinal regression. However, there is an inconsistency problem between different binary classifiers. Cao et al. proposed the CORAL framework, which solves the inconsistency problem in predictions by having binary classification tasks with the same weight parameters but independent bias units, reducing the complexity of the model [29]. In addition, there is another strategy, controlling the different weights of labels [30], which was originally a method to solve the problem of class imbalance. Similarly, a competitive weighting method is used to change the original one-hot encoding [31]. 

From another perspective, we can consider it to be a continuous stack of clear image scores. Due to the competitive mechanism of SoftMax, the true value frame has a score much higher than other frames. In this paper, we experimentally compare the effects of different ordinal regression loss functions on the ultimate optimization of the network.

## 3. Materials and Methods

This section presents the method we propose. Section 3.1 describes the process of data collection, labeling, and data augmentation; Section 3.2 discusses the focusing strategies employed in this paper, which include full-stack search and single-frame prediction; and Section 3.3 details the linear self-attention mechanism [32] network architecture and the soft label loss function [31] utilized in this paper.

### 3.1. Dataset

**Figure 3:**
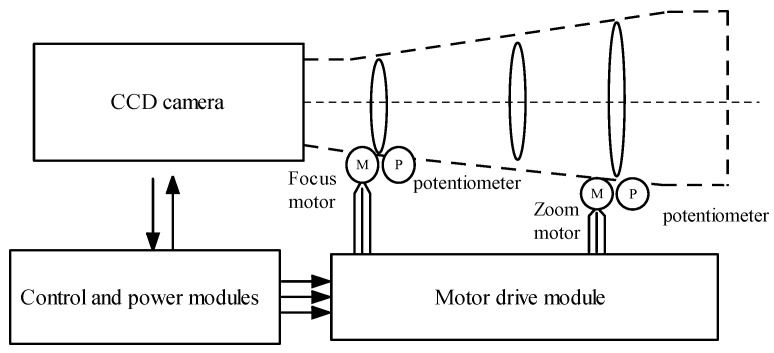
Lens focus control structure. The camera controls zoom and focus through the motor and uses a potentiometer to record the current position of the lens group.

Our dataset covers images from 51 different scenes captured through an optical lens. As shown in Figure 3, the autofocus process is a closed-loop control system that analyzes the images captured by the camera through the control module to determine the clarity of the image. If the image is not clear, the control module calculates the necessary adjustments for the lens group position and instructs the motor drive module to move the zoom motor or focus motor to adjust the lens group. Meanwhile, the potentiometer provides real-time feedback on the position of the lens group, and the control module makes iterative adjustments based on this feedback until the image is in the sharpest focus. The focal length of this lens is adjustable from 150 mm to 1500 mm, and the focus value potentiometer is adjustable from 200 to 960. 

**Figure 4:**
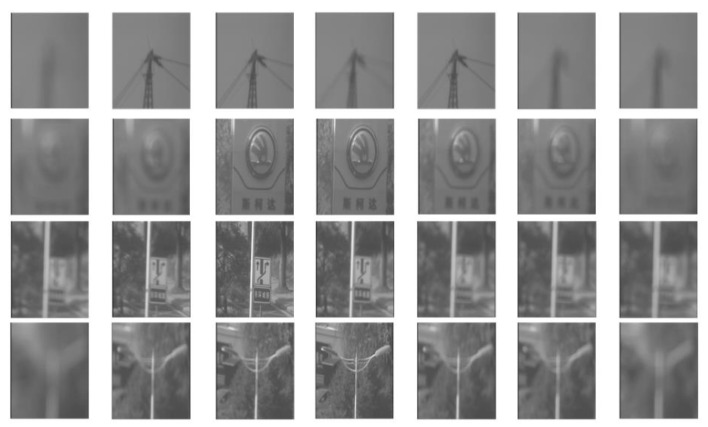
An image stack is a series of images obtained by continuously adjusting the focus value at a fixed focal length, and the stack includes the entire process of the images transitioning from blurry to sharp and back to blurry. Each image stack contains 300 to 400 images.

In order to obtain a rich set of image data, we fix a suitable focal length and generate a series of images with continuously varying focus values from 200 to 960 within a certain range by continuously adjusting the focus value of the lens. The characteristics of these continuous images transition from blurry to clear and then back to blurry. This series of continuously changing images captures the changes in the details of the scene, as shown in Figure 4. 

**Figure 5:**
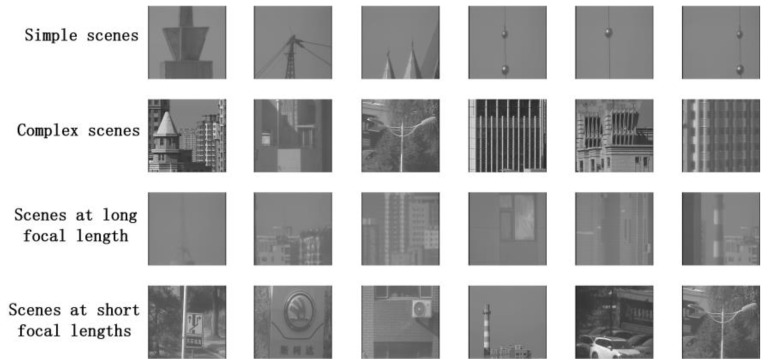
The dataset was collected under natural lighting conditions from 51 natural scenes, including simple scenes, complex scenes, scenes at a long focal length, and scenes at a short focal length, among other groups of data.

In Figure 5, we further demonstrate the various scenarios in the dataset, including a simple scene, a complex scene, a scene at a long focal length, and scenes at a short focal length.

To facilitate research, we standardize each image stack to ensure that the number of channels is fixed at 40, and all images are presented in grayscale. In addition, we also uniformly adjust the size of all images to 720 × 576 pixels for efficient computation and analysis. To determine the ground truth for each set of data, we adopt a combination of subjective and objective methods. The human eye can relatively quickly lock onto the range of images that are in focus, but it is not easy to distinguish between slightly out-of-focus and in-focus images. By using a method of observation by multiple people, we focus on evaluating the frame range where objects with clear prominence in the image are in focus. For the subtle differences between slightly out-of-focus and in-focus images, we further introduce a weighted wavelet function for precise judgment, thereby improving the accuracy of the ground truth.

(1) $$M=\alpha \cdot \sum_{\left(x,y\right)\in S_{{HL}_{4}}}w_{HL\left(x,y\right)}^{2}+\beta \cdot \sum_{\left(x,y\right)\in S_{{HL}_{4}}}w_{LH\left(x,y\right)}^{2}+\gamma \cdot \sum_{\left(x,y\right)\in S_{{HH}_{4}}}w_{HL\left(x,y\right)}^{2}\;$$

Here, $w_{HL\left(x,y\right)}^{2}$ represents the horizontal details of the image, $w_{LH\left(x,y\right)}^{2}$ represents the vertical details, and $w_{HL\left(x,y\right)}^{2}$ represents the diagonal details. α, β, and γ are the weights for each direction, respectively, and in this paper, the weights used are 0.4, 0.4, and 0.2, respectively. Subscript 4 represents the result after the fourth-order wavelet transform of the image. In actual testing, we use the “pywt.dwt2(img, h‘aar’)” function in Python to obtain the horizontal, vertical, and diagonal high-frequency coefficients of the “img” image. The parameter “haar” represents the wavelet basis function that we are using.

**Figure 6:**
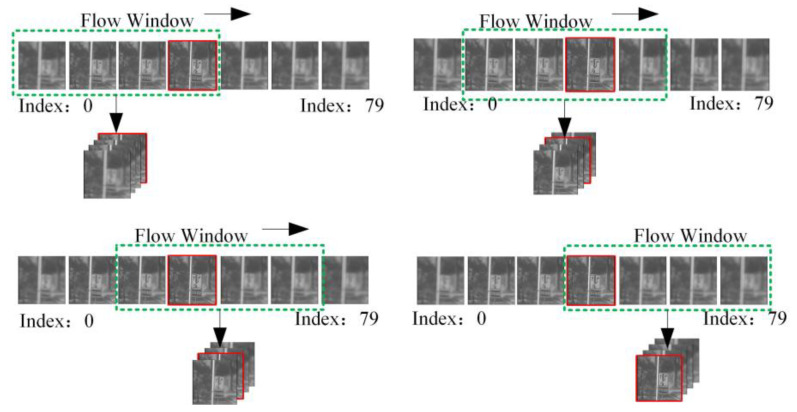
Sliding window method for data augmentation. The data are expanded by using a sliding window method, while ensuring that the ground truth is evenly distributed across the entire range of categories.

After determining the ground truth of the data, we find that the amount of data is small and the distribution of the ground truth is highly imbalanced, mainly concentrated within a few specific intervals. Due to the issue of having a small amount of data, we cannot use the cropping method for data augmentation, as this may lead to the loss of the main subject in the image; Moreover, the data imbalance is detrimental to the learning of the neural network, potentially causing the network to overfit to specific scenarios and thus affecting its generalization ability. Considering the particularity of the autofocus task, whether the image contains the main subject and the order corresponding to the image clarity is important to us, but the starting and ending numbers of the image sequence are not important. Therefore, we adopted a sliding window method for data augmentation. Taking into account that each focus stack contains 40 channels of data, we also set the length of the sliding window to 40. As shown in Figure 6, a stack of data with a length of 79 centered on the ground truth can be expanded into 40 sets of focus stack data, each with a length of 40, through the sliding window method. During this process, the position of the ground truth in each set of data is uniformly distributed between 0 and 39. With this data augmentation strategy, we not only enrich the dataset but also make the distribution of the ground truth more uniform across the dataset. This helps the neural network to better learn the characteristics of the focus stack data, improving its generalization ability and stability. In terms of dataset division, we set the ratio of the training set to the test set at 2:1. The training set contains 1360 image stacks, totaling 54,400 grayscale images, which are used to train the neural network model. The test set, on the other hand, contains 680 image stacks, totaling 27,200 grayscale images, which are used to evaluate the model’s performance. Through such data preprocessing and division, we provide a more balanced and enriched dataset for the training of the neural network, which is conducive to the model’s better learning and generalization. Table 1 presents the detailed information of our dataset, as well as the focal value range, focus range, and exposure methods of the optical lenses.

### 3.2. Autofocus Strategy

**Figure 7:**
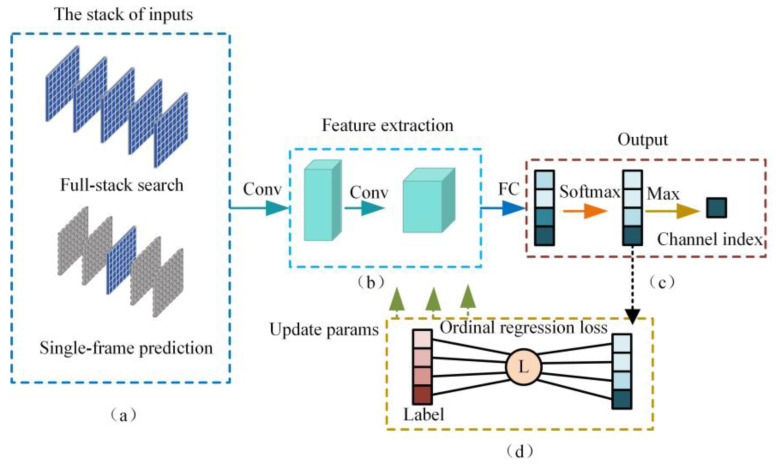
Full-stack search and single-frame prediction in autofocus. By training the full-stack search and single-frame prediction networks for two different focusing methods through a classification network, the network parameters are optimized using ordinal regression loss, which is different from the classification tasks in the past. (**a**) First, we modify the network’s input structure to multi-channel input, which is different from the general image processing problem that uses a three-channel RGB image input. We use the channel order to reflect the orderliness of the focus image sequence. The presence of data in the channels is determined based on full-stack search or single-frame prediction. (**b**) In the feature extraction part, we use existing classification networks to extract feature information from each channel, and classification networks have certain advantages in extracting the main targets in the image. (**c**) For the network output part, we prefer to use a classification-regression approach, rather than directly outputting the final channel number. The network’s output is the score of multiple channels, and the channel number with the highest score is obtained through softmax and max operations. (**d**) In the training process, choose the ordinal regression loss function corresponding to the ordinal regression problem. It was found in actual training that if only L1 smooth loss regression loss is used, the network’s convergence speed is very slow.

In this paper, we implement the autofocus function using full-stack search and single-frame prediction. We integrate a series of grayscale images with continuously varying focus values into a stack, mapping the focus values to the channel order of the stack. We use a classification network to treat the focusing issue as a classification problem, which avoids the output in fractional form. If we simply regarded autofocus as a classification problem, it would turn the focusing issue into a frame selection problem among a series of images, where the selection of the clearest image is required and the frame selection problem does not ensure the order of the data. Due to the specificity of the autofocus task in classification, we further introduce an ordinal regression loss function, transforming the classification problem into an ordinal regression problem. As shown in Figure 7, focusing networks have two methods of stacking inputs, and after extracting image features through convolutional layers, a classifier is used to output the channel index. During the training process, ordinal regression loss functions are employed to optimize the network parameters.

Full-stack searching is limited by the image acquisition rate because it requires the focusing motor to capture a complete image stack before performing the search. In contrast, the single-frame prediction method offers higher real-time performance. Compared to full-stack searching, the stack images used in single-frame prediction only provide the image currently captured by the focusing motor and the lens group position mapped to the channel index, resulting in a reduction in available information and an increase in the difficulty of focus prediction. For the single-frame prediction dataset, channel data randomly selected from the entire focusing stack are used as input data, while the other channels are set to zero, simulating the random initial position of the focusing motor in real-world focusing scenarios.

The focusing strategy we adopt has distinct advantages in two dimensions. Firstly, we are committed to solving the autofocus problem through an end-to-end deep learning approach. By processing the complete focus stack images synchronously with a convolutional network, we are able to automatically learn more accurate and effective features, thereby eliminating the traditional manual feature design process. Secondly, we achieve the network’s single-frame prediction capability by further reducing the required input information, particularly by reducing the input to a single-frame image and the channel index corresponding to the position of the focusing potentiometer.

### 3.3. Network Architecture and Loss Function

Thanks to the maturity of neural network research in the field of image classification, we can leverage a multitude of existing network models for testing. However, considering that the task scenarios are mostly embedded applications with high restrictions on computational resources and storage space, this paper conducted a detailed comparative verification of several mainstream lightweight networks. We attempt to explore the optimal coupling methods between several mainstream lightweight networks and different loss functions, in the hope of achieving the best balance of performance and computational efficiency. 

#### 3.3.1. Network Architecture

**Figure 8:**
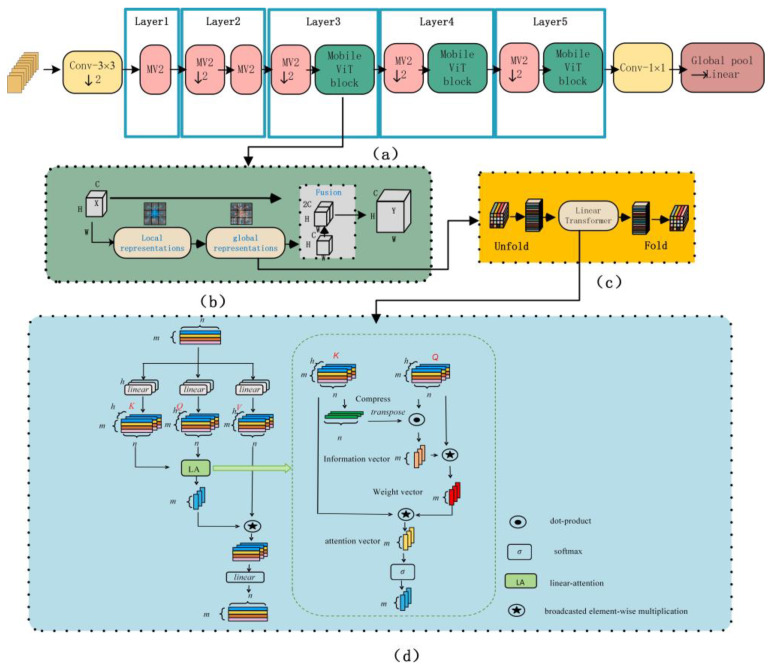
The structure of the model and linear self-attention. This figure shows the main structure of the classification network we used. We adjusted the network input to multi-channel input and modified the calculation method of the transformer in the global feature representation, introducing a self-attention mechanism with linear complexity, further reducing the computational load of the network. (**a**) “mv2” represents the mobilenetv2 block, which is an inverted residual structure. The internal structure of the MobileViT block is shown in (**b**). Its advantage lies in combining the spatial inductive bias of the convolutional network with the global inductive bias of the transformer structure, as shown in (**c**). The global feature is extracted through the “unfold-linear transformer-fold” method. The unfold operation refers to dividing the two-dimensional image or feature map into multiple small rectangular areas (patches), and then linearizing these areas into a one-dimensional form. The fold operation is the inverse process of unfold, which converts the serialized data back into a two-dimensional form. However, the introduction of the transformer self-attention mechanism increases the computational cost of the network. To this end, we introduced linear attention to reduce the network’s computing power requirements. The specific calculation method of the linear self-attention mechanism is shown in (**d**). The original self-attention mechanism fuses the attention feature map at the K and Q feature map, which we modified to calculate a set of attention vectors.

As shown in Figure 8, we modify the basic network architecture of MobileViT [33]. Firstly, the input and output structure of the original network are modified to adapt to the image stack input of this paper., The original MobileViT block introduces a linear attention mechanism, becoming the Linear MobileViT block [32], which enhances the network’s original global feature extraction capability while reducing computational complexity. To further reduce the classification error of the model, we combine the soft label cross-entropy loss function [31] after the classifier during the training phase. As shown in Figure 8a, the final network architecture includes five layers of feature extraction and uses the soft label loss function to optimize the parameters.

As shown in Figure 8b, the core feature extraction method of the MobileViT network [33] involves using convolutions to extract local features from the input image. Subsequently, global feature extraction is achieved through the “Linear Transformer” approach, and finally, the extracted features are fused with the original features.

As shown in Figure 8c, the core feature of the Linear MobileViT block [33] lies in its seamless integration of traditional convolutional feature extraction with the transformation capabilities of the transformer. However, the introduction of the transform module leads to a large computational load for the network. We introduce a self-attention mechanism [32] with linear complexity to reduce the computational complexity of the model.

As shown in Figure 8d, this mechanism generates a linear complexity attention vector by fusing the K (key) and Q (query) vectors. Initially, the K matrix is compressed along the patch dimension to obtain a set of vectors of length n. These compressed vectors are then used to fuse with the Q matrix to create an information vector. This information vector is used to normalize the information in the Q matrix, resulting in a weight vector for the K vectors. By fusing the K vectors with this weight vector, the final attention vector is obtained. This attention vector is then passed through a softmax competition mechanism to increase the weights of certain patches. Compared to the original self-attention matrix, the softmax here operates on a linear vector, greatly reducing the computational complexity. Ultimately, the attention vector is applied to the V (value) matrix, allowing for the feature re-extraction of the V matrix and differentiating the attention weights between various tokens, thus avoiding the issue of trivial attention.

#### 3.3.2. Loss Function

Next, let us discuss the loss function we adopt: In classification tasks, it is common to use one-hot encoded vectors to represent each class, where only one element in the vector is 1 and all other elements are 0. The position of this 1 corresponds to the true class. During training, classification loss functions such as cross-entropy are typically used, and the output layer of the neural network employs a softmax activation function to ensure that the network’s output and the true labels are both probability distributions. In this way, the network learns to simulate these one-hot encoded vectors as closely as possible, so that the class with the highest probability in the output layer matches the true class. In other words, the cross-entropy loss essentially selectively extracts the result from the network’s output. In scenarios with independent classes, the order of categories is not important, which is reflected in the one-hot encoded vectors. These vectors exclude any remote similarities that might exist between incorrect classes and the true class, ensuring that the position of the true class is emphasized and distinct from other incorrect classes. However, this approach is not suitable for ordinal regression problems because, in ordinal regression, some true labels may be more correct or closer to others.

**Figure 9:**
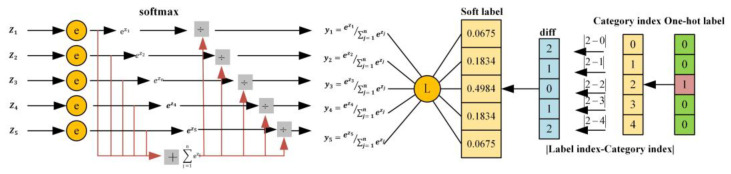
Soft label loss function. The diagram illustrates the process of calculating a loss function with soft labels for five categories. On the left side, $z_{1}$ to $z_{5}$ represent the outputs of the five nodes in the last layer of the neural network. Through softmax, we can enhance the competitive mechanism of the network, allowing categories with higher scores to obtain even higher scores, thus yielding the five outputs $y_{1}$ to $y_{5}$. In terms of label processing, we have abandoned the original one-hot encoding method. Instead, we first calculate the absolute difference between the index of the true value and each label. Based on the calculated absolute difference, we obtain the final soft label through Formula (2).

As shown in Figure 9, the strategy of using soft labels [31] to add ordinality to classification networks is to introduce a type of soft label that can easily represent the sequential nature of different levels without the need for multiple binary classifiers. Clearly, this approach makes a significant adjustment to the absolute authority of the true values represented by the original one-hot encoding, and it will largely sacrifice the accuracy of the network’s classification. This requires the network to strike a balance between error rate and accuracy.

We calculate a coded vector as a specific instance of our true value label *y* in:

(2) $$y_{i}=\frac{e^{-\varphi \left(r_{t},r_{i}\right)}}{\sum_{k=1}^{K}e^{-\varphi \left(r_{t},r_{k}\right)}}\;\;\;\forall r_{i}\in y$$

where $\varphi \left(r_{t},r_{k}\right)$ serves as a measure for the choice of the loss function, and in this paper, the L1 norm is used as the distance metric. Inspired by the competitive mechanism of softmax, a negative sign is introduced into the original exponential term. Under such a framework, the element closest to the true ordinal class (or match) will obtain the highest value, while other classes are assigned corresponding weights based on their distance from the true class. As the distance between an element and the true class increases, its value will gradually decrease.

## 4. Results

This section presents the experimental results and some comparative results of the method proposed in this paper. Section 4.1 below details the evaluation metrics used in this paper. Section 4.2 presents our experimental process and results. In Section 4.3, we discuss and analyze the overall experimental results.

### 4.1. Evaluation Metric

For the autofocus task, the most critical aspect we focus on is the accuracy of each focusing attempt, which is directly reflected in the error between the predicted frame serial number and the true frame serial number. Especially when dealing with a focus stack, this error value becomes a key metric for measuring the model’s performance. To fully and accurately assess this error, the current experiment employs the mean absolute error (MAE) and the root mean squared error (RMSE) as the calculation standards. In addition to the above error metrics, the running time of the algorithm is also an indispensable factor in evaluating its performance. An excellent algorithm not only needs to have high accuracy but also needs to complete the computation within a reasonable time to meet the requirements of practical applications.

Therefore, in this experiment, we comprehensively consider both the running time of the algorithm and the error metrics to provide a thorough evaluation of the model’s performance. Through this experiment, we hope to find an autofocus method that is both accurate and efficient, providing strong support for research and applications in related fields. At the same time, we also hope that these experimental results can provide valuable references and insights for future research.

#### 4.1.1. MAE

MAE, which stands for mean absolute error, represents the average of the absolute differences between predictions and observations. It is a linear score where all individual differences are weighted equally in the average. MAE is easy to understand because it directly calculates the average of the residuals. Compared to MAE, RMSE (root mean squared error) penalizes larger differences more.

(3) $$MAE\left(X,h\right)=\frac{1}{m}\sum_{i=1}^{m}\left|h\left(x_{i}\right)-y_{i}\right|$$

#### 4.1.2. RMSE

RMSE, which stands for root mean square error, is the sample standard deviation of the differences between predicted values and observed values, also known as residuals. The root mean square error is used to measure the extent of variation or dispersion of the sample.

(4) $$RMSE\left(X,h\right)=\sqrt{\frac{1}{m}\sum_{i=1}^{m}{\left(h\left(x_{i}\right)-y_{i}\right)}^{2}}$$

### 4.2. Experiments

The hardware configuration used in this experiment is the NVIDIA RTX 2080 Ti graphics card, which has excellent computational power and video memory capacity, providing strong hardware support for the training of deep learning models. During the training process, we set the batch size to 5 and we divide the dataset into a training set and a test set in a ratio of 2:1. During the training process, we employ the Adagrad optimization method, The learning rate is set to 0.0002. The pixel values of each channel are scaled to the range of [−1, 1].

#### 4.2.1. Comparative Experiment of Full-Stack Search

In this experiment, we specifically select image data that cover a variety of complex background conditions, including distant and close-up targets, which exhibit different levels of clarity and detail under different depths of field and backgrounds. Furthermore, to more comprehensively test the performance of sharpness evaluation functions, we also intentionally introduce targets with saturated speckles in grayscale, which pose a significant challenge to traditional sharpness evaluation functions. These targets, due to their special grayscale distribution and speckle effects, often make it difficult for traditional sharpness evaluation functions to accurately assess.

To more objectively verify the results of this experiment, we do not limit ourselves to using only a few sharpness evaluation functions. Instead, we extensively select 18 different sharpness evaluation functions from existing methods for comparative experiments in Table 2. These functions include traditional, gradient-based, and frequency domain-based, aiming to comprehensively assess their performance under different types of targets and background conditions. By comparing the performance of these functions on the same dataset, we can more clearly see their respective strengths and weaknesses, thereby providing valuable references for subsequent research and applications.

Traditional methods generally use global or specific local evaluations of the image. When facing targets with complex backgrounds and multiple depths of field, the image quality is often greatly affected by the background. In contrast, neural networks adaptively select the main subjects in the image as the primary objects for feature extraction, which is similar to the way humans understand an image.

#### 4.2.2. Experiments on the Synergy Optimization of Model and Loss Function

In order to explore the optimal loss function for autofocus, we compare a variety of models combined with three classic loss function strategies, namely the traditional cross-entropy loss function, the COPAL loss function [29], and the soft label loss function [31]. We compare the training methods of each model’s different depth versions combined with different loss functions, with the expectation of finding the most suitable network and loss function for this task.

**Figure 10:**
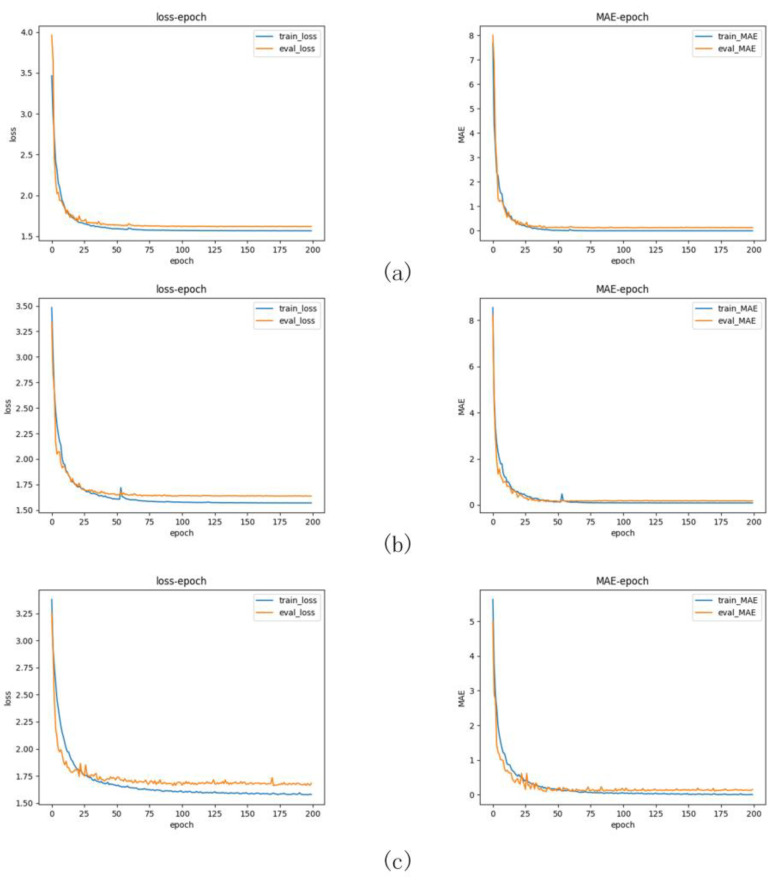
Training curves and error curves. In the figure above, (**a**–**c**) represent the training curves and error curves for the third-best, second-best, and the best results, respectively.

In Table 3, the red, blue, and green markings represent the best, second-best, and third-best results, respectively. Neural networks, due to their inherent hardware advantages, outperform traditional algorithms in terms of algorithm time; in terms of error, it can be observed through comparison that the training results of our model combined with the three loss functions are all optimal in performance, among which the training combined with the soft label [31] yield the best results, with a MAE of 0.094 and a RMSE of 0.201. Figure 10a–c represent the training curves and error curves for the third-best, second-best, and the best results, respectively. In Table 4, we compare the training effects of other methods under different loss functions.

#### 4.2.3. Self-Attention Mechanism Efficiency and Visualization Effect Experiment

The comparison table of model computational power and parameter count in this paper is shown in Table 5 with the following data all tested using an input tensor of 720 × 576 *×* 40. Compared to baseline MobileViT-xxs, our network increased the parameter count by 0.1 M but reduced computational power by 54%.

**Figure 11:**
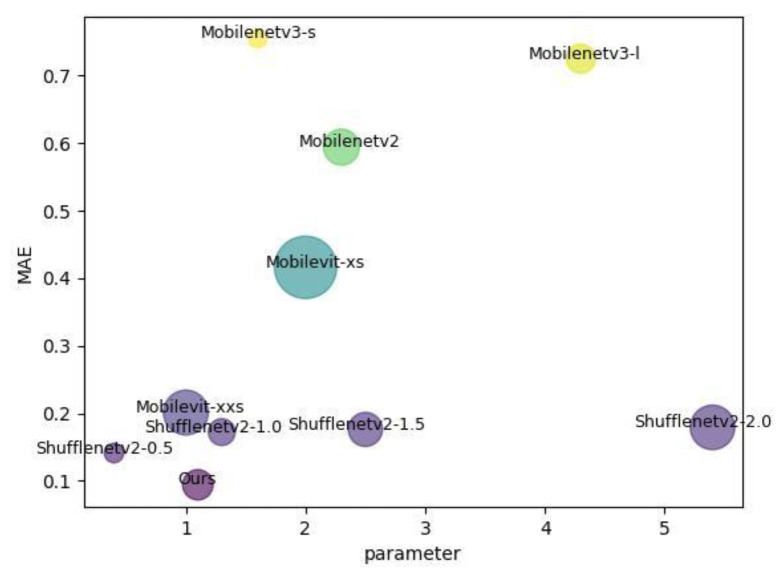
Parameter amount, error, and GFLOPS bubble chart. In the above figure, the closer the bubble is to the coordinate origin, the better the performance; the smaller the area of the bubble, the less computational resources are required. It can be seen that the comprehensive performances of our algorithm, shufflenetv2-0.5, and shufflenetv2-1.0 are better than those of other models.

In Figure 11, we display the parameters and comprehensive performances of each model using a bubble chart.

**Figure 12:**
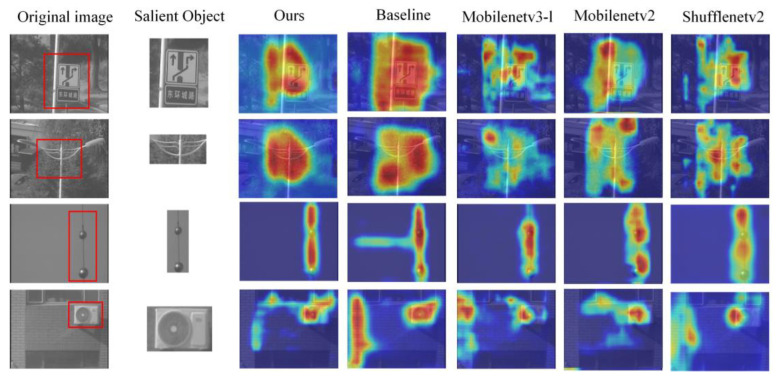
The first column of red boxes in the figure represents the salient target areas in the original image. In the heat map, the more sensitive areas have higher temperatures, which are represented by deeper reds, while the less sensitive areas have lower temperatures, represented by deeper blues. Our network’s region of interest is dynamically changing, and it focuses more on the main subject in the frame compared to other networks.

As shown in Figure 12, focusing methods that use neural networks are generally capable of extracting features of salient targets in the scene. Whether it is for complex backgrounds or single targets, the region of interest identified by neural networks is focused on the target. In comparison, the network modified in this paper has the ability to select a more concentrated and accurate region of focus.

#### 4.2.4. Comparative Experiment of Single-Frame Prediction

Based on the test results of full-stack searching, we select 10 training methods that combine the models with better training results and loss functions mentioned above to train the single-frame prediction network. As shown in Table 6, it can be observed that after reducing the input volume of the network, the prediction error increased to some extent, but the error is still within an acceptable range. This indicates that the neural network can determine the uncertainty of the focus direction on both sides by using the information from a single frame and the channel information, thereby outputting an accurate focus channel number. It has also been found that this channel encoding method results in the same running time as the full-stack search algorithm, with the advantage of not needing to capture the entire image stack. It minimizes the number of turns of the focusing motor to zero, further enhancing the focusing accuracy.

### 4.3. Result Visualization

**Figure 13:**
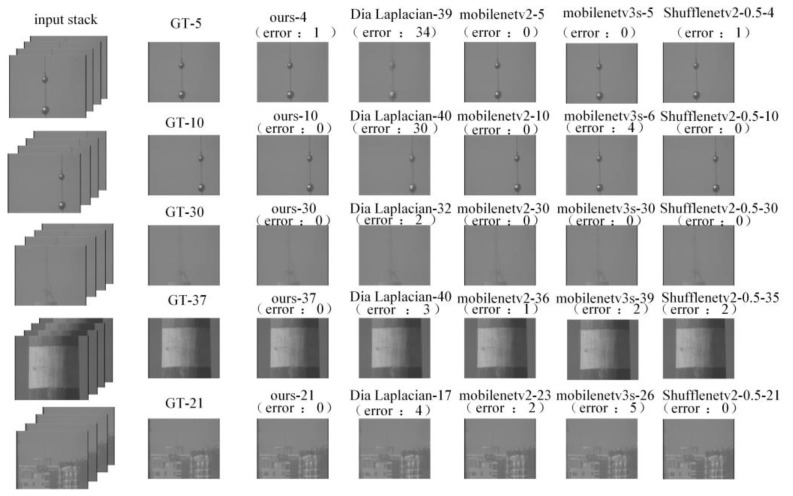
Focus results.

As shown in Figure 13, We demonstrate the difference in focusing effects between using a neural network and traditional evaluation functions under the full-stack search for difficult samples. It can be observed through comparison that traditional evaluation functions often fail to achieve focus on spot samples and struggle to distinguish between clear and slightly clear differences in low-contrast data, whereas neural networks can achieve precise focusing in these scenarios through training.

## 5. Discussion

Regarding the construction of the dataset, there is still much room for improvement on the basis of this paper. For example, collecting more scenes to reduce the impact of semantic overlap on the network, while also improving the model’s generalization performance.

In ordinal regression tasks, the choice of network depth has a crucial impact on model performance. A network that is too deep may lead to overfitting during the training process, which means the model performs well on the training data but poorly on unseen test data. This is because a network that is too deep can easily capture noise and irrelevant details in the training data, rather than learning the true data distribution. Therefore, there is an optimal depth threshold for different network architectures that allows the model to achieve good performance on both training and test data. To find this threshold, a series of experiments and adjustments are usually required.

In the process of selecting an appropriate loss function for our model, we conduct a comparative analysis of several prevalent loss functions, including cross-entropy, CORAL [29], and soft labels [31]. The objective is to identify a loss function capable of significantly reducing the mean prediction error, even if it meant compromising to some extent on the classification accuracy. Upon conducting a series of experiments and evaluating the comparative outcomes, it is determined that the soft label loss function [31] exhibited the most superior performance for the task at hand.

The methodology for single-frame prediction necessitates the amalgamation of data from a single image frame along with the channel position at which this frame is situated. In the current work, we employ a technique that involves nullifying the information from all channels except the one of interest. This approach, to some extent, results in the inefficient utilization of computational resources, as it augments the model’s parameter count and extends the time required for prediction. In our ongoing and future research endeavors, we aim to investigate alternative, more efficient means of inputting data for single-frame predictive models to mitigate these drawbacks.

## 6. Conclusions

To encapsulate our findings, we developed a dataset specifically designed for the purpose of autofocus in imaging systems. Concurrently, we introduced a lightweight autofocus method that leverages full-image stack inputs. This full-image input strategy, despite the trade-off of utilizing more computational resources, streamlined the process by bypassing the step of manual focus selection. We conducted experiments with two distinct focusing strategies—full-stack search and single-frame prediction—and both demonstrated improvements over conventional autofocus techniques. At the core of our network’s architecture, we integrated a linear attention mechanism, which significantly boosted the network’s efficacy and led to a reduction in computational demands by 54%. In addressing the ordinal regression aspect of autofocus, we conducted a thorough comparison of prevalent loss functions when paired with various network configurations. This analysis culminated in the selection of the network structure and the soft label loss function [31] as the optimal combination for our study. We are confident that the insights presented in our paper offer a novel perspective on autofocus technology and anticipate that it will serve as a beneficial reference for future research endeavors and practical applications in the field.

## Figures and Tables

**Table 1:** Detailed information and technical specification on the optical lens used.

Focal Value Range	Focus Value Range	Exposure Mode	Illumination	Resolution	Number of Scenes	Training Set	Evaluation Set
160 mm–1500 mm	200–960	automatic exposure	natural lighting	720 × 576	51	1360	680

**Table 2:** Comparative experiment between full-stack search and traditional evaluation functions.

Inxdex	Algorithm	MAE	RMSE	T/ms
1	Histogram Entropy [2]	19.500	22.661	188
2	DCT Reduced Energy Ratio [13]	16.897	19.730	248
3	Percentile Range [2]	6.905	7.581	90
4	Wavelet Variance [11]	6.900	8.979	352
5	Modified DCT [3]	6.884	8.617	48
6	Wavelet_Ratio [11]	6.645	8.216	357
7	Gradient Magnitude Variance [4]	6.586	8.135	350
8	Intensity Coefficient of Variation [2]	6.546	7.360	110
9	DCT Energy Ratio [2]	6.499	7.348	250
10	Intensity Variance [2]	6.419	7.322	90
11	Total Variation L2 [34]	5.652	6.234	100
12	Gradient Count [2]	5.634	6.279	240
13	Mean Gradient Magnitude [35]	5.603	6.082	345
14	Laplacian Variance [4]	5.110	8.148	85
15	Total Variation L1 [34]	4.559	5.091	130
16	Wavelet Sum [11]	3.987	5.240	340
17	Wavelet Weighted [11]	3.289	3.589	317
18	Diagonal Laplacian [36]	1.619	6.408	240
19	Ours	0.094	0.201	28

The red, blue, and green markings represent the best, second-best, and third-best results, respectively.

**Table 3:** Comparative experiments with neural networks combining different loss functions.

Backbone Network	Cross-Entropy			CORAL [29]		Soft Label [31]			
	MAE	RMSE	T/ms	MAE	RMSE	T/ms	MAE	RMSE	T/ms
Mobilenetv3-l [37]	1.857	3.661	18.6	0.755	1.456	18.5	0.971	1.903	19.1
Mobilevit-xs [33]	0.707	1.353	25.9	0.416	0.684	25.9	0.179	0.358	25.8
Mobilevit-xxs [33]	0.588	0.823	24.2	0.567	0.803	22.6	0.201	0.397	23.7
Shufflenetv2-2.0 [38]	0.732	1.445	18.4	0.837	1.395	19.7	0.179	0.354	18.3
Shufflenetv2-1.5 [38]	0.694	1.331	18.1	0.456	0.877	19.8	0.176	0.341	18.8
Shufflenetv2-1.0 [38]	0.572	0.823	18.7	0.232	0.435	18.6	0.172	0.327	18.4
Shufflenetv2-0.5 [38]	0.498	0.782	18.5	0.238	0.449	18.4	0.141	0.284	18.2
Ours	0.296	0.589	27.5	0.173	0.328	29.0	0.094	0.201	27.8

The red, blue, and green markings represent the best, second-best, and third-best results, respectively.

**Table 4:** Comparative table of other ML methods.

Method	Cross-Entropy			CORAL [29]		Soft Label [31]			
	MAE	RMSE	T/ms	MAE	RMSE	T/ms	MAE	RMSE	T/ms
Herrmann [2]	1.731	3.551	17.4	0.594	1.186	17.7	1.231	2.164	17.6
Liao [21]	2.439	4.889	17.5	0.725	1.433	17.3	1.477	2.293	17.2
Ours	0.296	0.589	27.5	0.173	0.328	29.0	0.094	0.201	27.8

The red, blue, and green markings represent the best, second-best, and third-best results, respectively.

**Table 5:** Comparison table of model computational power and parameter count.

Model	GFLOPS	Parameter/M
Mobilenetv2 [39]	3.8	2.3
Mobilenetv3-l [37]	2.5	4.3
Mobilenetv3-s [37]	1.0	1.6
Mobilevit-xs [33]	11.4	2.0
Mobilevit-xxs [33] (baseline)	5.9	1.0
Shufflenetv2-2.0 [38]	5.8	5.4
Shufflenetv2-1.5 [38]	3.4	2.5
Shufflenetv2-1.0 [38]	2.1	1.3
Shufflenetv2-0.5 [38]	1.1	0.4
Ours	2.7	1.1

Red represents the parameters of the modified model, and blue represents the parameters of the baseline model.

**Table 6:** Comparative table of single-frame prediction.

Model and Loss Function	MAE	RMSE	T/ms
Mobilenetv2 [39] CORAL [29]	0.611	1.212	17.6
Mobilenetv3-s [37] CORAL [29]	0.811	1.561	17.4
Mobilenetv3-l [37] CORAL [29]	0.651	1.312	18.7
Mobilevit-xs [33] Soft label [31]	0.246	0.478	25.7
Mobilevit-xxs [33] Soft label [31]	0.232	0.463	23.8
Shufflenetv2-2.0 [38] Soft label [31]	0.229	0.441	18.6
Shufflenetv2-1.5 [38] Soft label [31]	0.223	0.432	18.4
Shufflenetv2-1.0 [38] Soft label [31]	0.181	0.352	18.2
Shufflenetv2-0.5 [38] Soft label [31]	0.178	0.334	18.2
Ours Soft label [31]	0.142	0.293	27.5

The red, blue, and green markings represent the best, second-best, and third-best results, respectively.

## Data Availability

Data underlying the results presented in this paper are not publicly available at this time but may be obtained from the authors upon reasonable request.
